# Parametric Imaging for Precision Medicine: Our Perspective on the Special Issue “Applications of Imaging Technology in Human Diseases”

**DOI:** 10.3390/biomedicines14020277

**Published:** 2026-01-27

**Authors:** Giulio Distefano, Antonio Granata

**Affiliations:** 1 Institute of Nephrology and Dialysis, Maggiore Hospital of Modica, ASP Ragusa, 97015 Modica, Italy; 2 Nephrology and Dialysis Unit, Cannizzaro Hospital, 95100 Catania, Italy; antonio.granata4@tin.it

## 1. Introduction

This Special Issue was born from a simple yet powerful observation: imaging is no longer only about seeing, but also about measuring. Modern imaging has become a platform capable of quantifying biological phenomena, thereby supporting clinical decisions and guiding interventions in a reproducible manner. Quantification transforms signals into numbers that can be compared across time and centers, fostering consistency and reliability in patient care. Although this Special Issue was not specifically devoted to these themes, we are convinced that, in the near future, imaging will inevitably be shaped by three converging directions: the technological leap in terms of hardware, which continuously expands the boundaries of what can be visualized; the parametric nature of new generation imaging—such as quantitative magnetic resonance imaging (qMRI), contrast-enhanced ultrasound (CEUS), and elastography—capable of translating biological signals into objective metrics; and the standardization of biomarkers and reporting, including the indispensable integration of artificial intelligence (AI). Together, these directions outline an unavoidable paradigm shift that will redefine imaging’s role in precision medicine. The intent of this Special Issue was to highlight, once again, how imaging can be essential in precision medicine. Across the contributions we selected, a consistent message emerges: imaging generates value when measurements are anchored to a clear use context and demonstrably influence patient management

## 2. Hardware Matters in Everyday Practice

Furthermore, new hardware platforms are raising the bar. For example, photon-counting computed tomography (PCCT) is no longer a cutting-edge technique but has become part of diagnostic routine, offering higher spatial resolution, noise reduction, and improved contrast resolution while maintaining very favorable dosimetry compared with previous-generation machines [1,2]. These features are not merely technical: resolving small structures—especially in vascular imaging—affects diagnosis and procedural planning [1,2]. For us, hardware is clinically relevant only when its quantitative output changes what we do next (therapy adjustments or the need for further investigations). This concept can be extended to all other diagnostics used daily in the clinical setting, as is evident from the contributions examined.

## 3. From Images to Parameters: qMRI, CEUS, and Elastography

Fundamentally, parametric imaging is quantitative, not qualitative. In nephrology, qMRI has introduced fascinating techniques, providing complementary windows into renal physiology. These include arterial spin labeling (ASL) for perfusion; blood oxygenation level-dependent (BOLD) imaging as an indirect proxy of tissue oxygenation/hypoxia; diffusion-weighted imaging and the apparent diffusion coefficient (DWI/ADC) for tissue diffusivity; and T1/T2 mapping for tissue characterization. Together, these parameters enable a multiparametric assessment of both native and transplanted kidneys [3,4]. These parametric techniques are promising because they link tissue signal to relevant pathophysiological characteristics (hemodynamics, hypoxia, fibrosis) and are suitable for longitudinal monitoring and possibly for risk stratification [3,4]. Initially centered on focal liver lesions, parallel CEUS now extends to extrahepatic and vascular applications. Recommendations from the European Federation of Societies for Ultrasound in Medicine and Biology (EFSUMB) standardize acquisition, analysis, and reporting for perfusion assessment, improving comparability across studies and centers. In renal transplantation, elastography—and in particular shear-wave elastography (SWE)—represents a virtuous bridge between imaging and histopathology. Quantitative stiffness correlates with interstitial fibrosis and tubular atrophy, lending itself to longitudinal monitoring. We use SWE to track trends: it does not replace biopsy, but serial measurements can flag fibrotic trajectories in cohorts and prompt closer review in individual patients when values deviate meaningfully from the ranges reported in the literature [5]. CEUS-derived perfusion metrics complement stiffness by adding microvascular information within codified protocols [6].

## 4. From Parameters to Biomarkers

The clinical value lies not only in mere morphological assessment but also in the availability of “imaging biomarkers” with robust levels of evidence suitable for supporting decisions.

The European Imaging Biomarker Alliance (EIBALL) of the European Society of Radiology (ESR) offers a structured overview of accredited biomarkers and their clinical correlations [7,8]. The Radiological Society of North America (RSNA) Quantitative Imaging Biomarkers Alliance (QIBA) develops profiles that specify how to acquire, process, and report data to achieve measurement accuracy and reproducibility suitable for clinical use and multicenter research [9]. In our view, these frameworks are the scaffolding that turns parameters into decisions.

## 5. AI Reporting Frameworks and Designing Studies

AI is permeating imaging. To limit bias and over-interpretation, trials that incorporate AI should follow SPIRIT-AI (Standard Protocol Items: Recommendations for Interventional Trials–Artificial Intelligence) and CONSORT-AI (Consolidated Standards of Reporting Trials–Artificial Intelligence) [10,11]. For predictive models, TRIPOD-AI (Transparent Reporting of a multivariable prediction model for Individual Prognosis Or Diagnosis–Artificial Intelligence) provides a common reporting grammar—dataset characteristics, feature handling, preprocessing, external validation, and calibration—so that in silico performance can be judged in clinically meaningful terms [12].

## 6. What This Collection Shows

In the context of this Special Issue, we welcomed both original research and reviews across oncology, cardio-neuroradiology, musculoskeletal imaging, and nephro-radiology, including AI-enabled workflows and procedural use cases. A recurring element links these papers: quantitative metrics—perfusion parameters, stiffness estimates, spectral indices—are used to make care more efficient, by avoiding redundant examinations or limiting biopsies to selected cases, for example. The topic heterogeneity is intentional; the direction is convergent—toward measurability, integration with care pathways, and outcomes—consistent with the overview on the Special Issue’s web page [13,14,15,16,17,18,19,20,21,22,23,24,25].

## 7. Focus on Nephrology and Transplantation

Nephrology offers an ideal testbed for imaging-based precision medicine. qMRI makes it possible to overcome the limitations of conventional functional indices derived from laboratory data on overall renal function in terms of indirect filtration capacity estimation (creatinine, clearance, eGFR). It does this by providing spatially resolved maps of perfusion, diffusion, and oxygenation, capable of assessing the activity of individual kidneys [3,4]. This is crucial in renal transplants, where early fibrosis and hypoxia identification can change management. Elastography, and in particular SWE, offers a reproducible stiffness measure linked to interstitial fibrosis and tubular atrophy; while it cannot, at present, completely replace biopsy, it supports serial follow-up and may predict dysfunction when values diverge from the published ranges [5]. CEUS contributes complementary perfusion indices within established protocols [6]. The unifying principle is parametricity: transforming signals into comparable numbers that can be trusted within and across centers in order to integrate imaging into both diagnostic and therapeutic pathways and into scientific research for codifying study protocols.

## 8. Conclusions

If there is a univocal message, it is that imaging produces value when its measurements become biomarkers with a clear use context for impacting decisions. Much has already been implemented, such as descriptors of biomarker validation and reproducible profiles [8,9], as well as assistance from emerging artificial intelligence in study structuring [10]. This Special Issue has gathered contributions in which imaging, whether innovative or revisited in protocols, has been applied in its most useful form, namely measurable, to respond to a clinical need, potentially limiting invasive procedures to selected cases. Imaging is the lingua franca of precision medicine: we hope to use it rigorously and in the correct context.
